# Non-invasive vagus nerve stimulation attenuates the burnout

**DOI:** 10.1192/j.eurpsy.2022.1896

**Published:** 2022-09-01

**Authors:** S. Tukaiev, O. Pravda, N. Vysokov, A. Tarasenko, D. Toleukhanov, V. Komarenko, S. Danylov, V. Kravchenko

**Affiliations:** 1 National Taras Shevchenko University of Kyiv, Institute Of Biology And Medicine, Kyiv, Ukraine; 2 BrainPatch Ltd, Brainpatch Ltd, London, United Kingdom; 3 Beehiveor Academy and R&D Labs, Beehiveor Academy And R&d Labs, Kyiv, Ukraine

**Keywords:** EEG, burnout, neuromodulation, vagus nerve stimulation

## Abstract

**Introduction:**

Vagus nerve stimulation produces therapeutic effects for the treatment of psychiatric disorders, heart failure, and others.

**Objectives:**

The aim of the current study was to evaluate the effects of the non-invasive vagus nerve stimulation on emotional burnout.

**Methods:**

6 right-handed male volunteers aged 18-22 years participated in study. We used the combination of pleasant meditative classical music and a slow bi-polar wave (0.1-0.2 Hz) of electrical non-invasive transcutaneous auricular vagus nerve stimulation for 5 minutes. The set of 4 VNS was performed at intervals of 3 days. EEG was registered during the rest state (3 min, closed eyes condition). To measure the severity of emotional burnout in students, we used the 22-item Maslach Burnout Inventory (MBI).

**Results:**

VNS significantly improve the depersonalization and reduction of personal achievements (components of the emotional burnout). Changes in the psychoemotional state of the respondents were accompanied by the increase in the theta-Fz/alpha-Pz ratio, that reflects an enhancement of the activation level. A set of non-invasive stimulation of the auricular branch of the vagus nerve leads to an increase in the level of activation (the ratio of beta / alpha rhythms). The changes in the power of the alpha rhythm may relate to improving of mental process, creativity, creative thinking. An increase in alpha rhythm may reflect internally oriented attention in creative activities.

**Conclusions:**

The preliminary data suggests that the novel mastoid stimulation device may have a prolonged stimulating effect on the brain processes while attenuating the burnout at the same time.

**Disclosure:**

No significant relationships.

